# Different Bleeding Patterns with the Use of Levonorgestrel Intrauterine System: Are They Associated with Changes in Uterine Artery Blood Flow?

**DOI:** 10.1155/2014/815127

**Published:** 2014-04-23

**Authors:** Carlo Bastianelli, Manuela Farris, Stefania Rapiti, Roberta Bruno Vecchio, Giuseppe Benagiano

**Affiliations:** ^1^Department of Gynaecology, Obstetrics, and Urology, Sapienza University of Rome, Viale del Policlinico 155, 00161 Rome, Italy; ^2^C/O Associazione Italiana Educazione Demografica (AIED), Via Toscana 30, 00187 Rome, Italy

## Abstract

*Objective*. Evaluate if different bleeding patterns associated with the use of the levonorgestrel intrauterine system (LNG-IUS) are associated with different uterine and endometrial vascularization patterns, as evidenced by ultrasound power Doppler analysis. *Methodology*. A longitudinal study, with each subject acting as its own control was conducted between January 2010 and December 2012. Healthy volunteers with a history of heavy but cyclic and regular menstrual cycles were enrolled in the study. Ultrasonographic examination was performed before and after six months of LNG-IUS placement: uterine volume, endometrial thickness, and subendometrial and myometrial Doppler blood flow patterns have been evaluated. *Results*. A total of 32 women were enrolled out of 186 initially screened. At six months of follow-up, all subjects showed a reduction in menstrual blood loss; for analysis, they were retrospectively divided into 3 groups: normal cycling women (Group I), amenorrheic women (Group II), and women with prolonged bleedings (Group III). Intergroup analysis documented a statistically significant difference in endometrial thickness among the three groups; in addition, mean pulsatility index (PI) and resistance index (RI) in the spiral arteries were significantly lower in Group I and Group III compared to Group II. This difference persisted also when comparing—within subjects of Group III—mean PI and RI mean values before and after insertion. *Conclusions*. The LNG-IUS not only altered endometrial thickness, but—in women with prolonged bleedings—also significantly changed uterine artery blood flow. Further studies are needed to confirm these results and enable gynecologists to properly counsel women, improving initial continuation rates.

## 1. Introduction

The levonorgestrel-releasing intrauterine system (LNG-IUS) has proven its efficacy, both as a long-acting contraceptive [1–4] and for its noncontraceptive benefits [5–7], including high effectiveness in the treatment of heavy menstrual bleeding [8–11]. However, prolonged or irregular bleeding during the first months after placement has been reported by 22% and 67% of women, respectively, usually declining by the end of the first year [12].

To reduce discontinuations due to the so-called “bleeding nuisances” [13, 14], women wishing to use the LNG-IUS should be properly counseled concerning bleeding patterns to be expected [15].

The mechanisms involved in these unexpected patterns remain unclear [8]. A study of endometrial biopsies from LNG-IUS users explored the local effect on the endometrium and documented significant modifications in endometrial vascularization, with a decrease in mean vascular density and an increase in mean vessel area [16]. An increase in subendometrial vascularization has also been documented in women complaining of major side effects (dysmenorrhea and/or irregular bleeding) following insertion of copper-releasing intrauterine devices (Cu-IUDs) [17, 18].

A Doppler flow sonographic analysis did not reveal any significant change in uterine artery flow between Cu-IUD or LNG-IUS users [19]; a marked reduction in the subendometrial blood flow and endometrial thickness was reported only in the LNG-IUS users. This phenomenon correlates well with morphological changes in the endometrial spiral arteries and capillaries reported in other studies [20, 21].

In light of previous work, we have repeated the power Doppler analysis of uterine and endometrial blood flows before and after placement of the LNG-IUS. In addition, to better understand possible influences of the system on uterine and endometrial vascularization, we have attempted to correlate results with various bleeding patterns occurring after insertion.

## 2. Methods

This is a longitudinal study, with each subject acting as its own control; the study was conducted between January 2010 and December 2012 at the Family Planning Centre, Department of Obstetrics, Gynecology and Urology, University La Sapienza, Rome, Italy. The study protocol was approved by the University Ethical Review Committee and was carried out following the principles of the Declaration of Helsinki. All participants provided written, informed consent before entry into the study and after the research protocol was explained in detail verbally.

Healthy women volunteers aged 18–45 years, with a history of regular cycles with heavy menstrual bleedings (cycle length within 21–35 days and menstrual blood loss >80 mL per menstruation, as determined by the Higham Pictorial Blood Loss Assessment Chart (PBAC)) and normal cervical smear, were recruited to the study.

Ultrasonographic examination was performed before LNG-IUS placement and six months after insertion with a vaginal probe 5 MH_2_ (Voluson E6 General Electric Ultrasound System), during midluteal phase, 6 to 9 days after ovulation, confirmed by ultrasound. All examinations were carried out by the same investigator, to avoid interobserver variation, and performed between 8.00 and 10.00 am.

Women were excluded if they were pregnant (a pregnancy test was performed in all subjects before recruitment) or breastfeeding, if they had a vaginal or caesarean delivery or abortion within 6 weeks of initial screening, and if they had a septic abortion or a postpartum endometritis within 90 days of screening. Subjects were also excluded in the presence of any uterine cavity distortion, past pelvic inflammatory disease, Chlamydia infection, pelvic tuberculosis, or malignancy.

The LNG IUS was then placed within 7 days of the onset of menstruation.

All women were given diary cards to record daily any vaginal bleeding. Bleeding and spotting days and episodes were assessed using the World Health Organization reference period developed by Belsey and Farley [22]. Bleeding intensity was scored according to the PBAC score (a score >100 was equivalent to blood loss >80 mL), before entering the study and after 6 months.

The three diameters (*D*) of the uterus were measured without including the cervix, and uterine volume was then calculated using the formula for an ellipsoid mass (4/3 × *π* × *D*1 × *D*2 × *D*3).

In order to evaluate uterine arteries flow patterns, along with morphological changes in the endometrium in subjects with different bleeding patterns, uterine artery's blood flow velocity waveforms on both sides were evaluated at the level of the inner cervical os; the subendometrial blood flow power Doppler analysis and endometrial thickness measurements were also carried out. The latter was measured as the thickest part in the sagittal section, including both endometrial layers, during the midluteal phase of the menstrual cycle.

Subendometrial blood flow has been assessed utilizing power Doppler energy (PDE) measurement and classified into five categories according to the subendometrial signal area percentage: I (<10%), II (10–25%), III (25–50%), IV (50–75%), and V (>75%), as described by others [23].

The Doppler gate was positioned as soon as a vessel with good color signal was obtained and then blood flow velocity waveforms were recorded. The pulsatility (PI: systole-diastole) and the resistance (RI: systole-diastole/systole) indices of both uterine arteries were calculated from the mean of three similar consecutive waveforms of good quality. The PI and RI values obtained from each artery were then averaged.

A statistical analysis was performed using SPSS (version 15, Chicago, IL, USA). For quantitative variables, the range, mean, and standard deviation were calculated. The difference between two means was statistically analyzed using the two-sided nonpaired* t*-test. The chi-square test was used for categorical data of subendometrial vascularization (power Doppler). A *P* value of 0.05 was considered statistically significant.

## 3. Results

**Figure 1:**
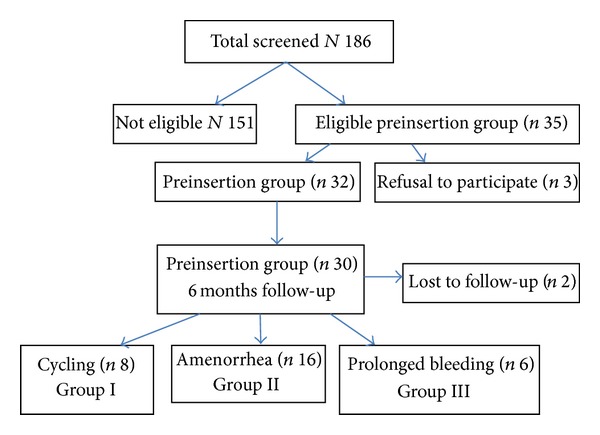
Study flow of participating women.

Out of a total of 186 women screened, 35—all with heavy menstrual bleedings—accepted to participate in the study and 32 were selected after full screening. Two additional subjects were excluded from analysis because of loss to follow-up (Figure 1).

The mean age of women enrolled in the study was 37.3 (range 25–46) years; all had at least a high school degree with a mean PBAC score of 338 (±182) (Table 1).

Clinically, at six months of follow-up, there was a reduction in menstrual blood loss for all women. Depending on the observed bleeding patterns, for the analysis, participants were divided into 3 groups. Group I included women in whom a cyclical menstrual bleeding pattern was maintained throughout the observation period. Group II comprised women who became amenorrheic after insertion of the system. Group III was made up of women with prolonged or irregular bleedings during treatment.

The three groups were analyzed separately in terms of uterine volume, endometrial thickness, subendometrial blood flow, PI, and RI and no significant association was found between a woman's obstetrical history, Body Mass Index (BMI), smoking habits, and cycle patterns.

**Figure 2:**
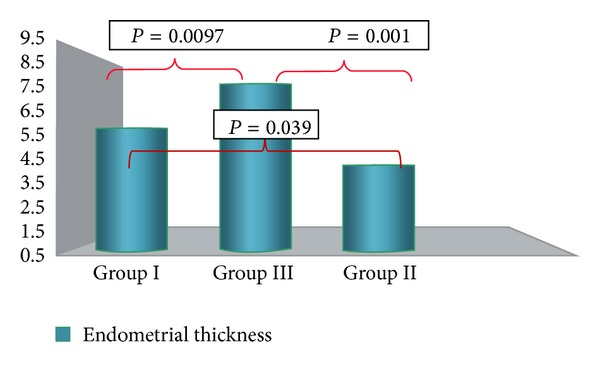
Intergroup analysis of endometrial thickness six months after insertion. A significant difference was observed between Group I (normal cycling women), Group II (amenorrheic), and Group III (prolonged bleedings).

An intergroup analysis disclosed a statistically significant difference in endometrial thickness (Figure 2), while uterine size and subendometrial blood flow did not differ. An additional statistically significant difference was found in endometrial thickness before and after insertion, but only in Group III (Table 2).

**Figure 3:**
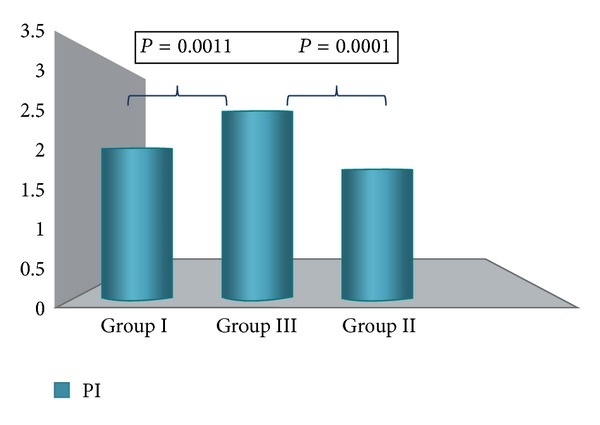
Mean PI values at six months of follow-up (intergroup analysis). A significant difference was observed between Group I (normal cycling women), Group II (amenorrheic), and Group III (prolonged bleedings).

**Figure 4:**
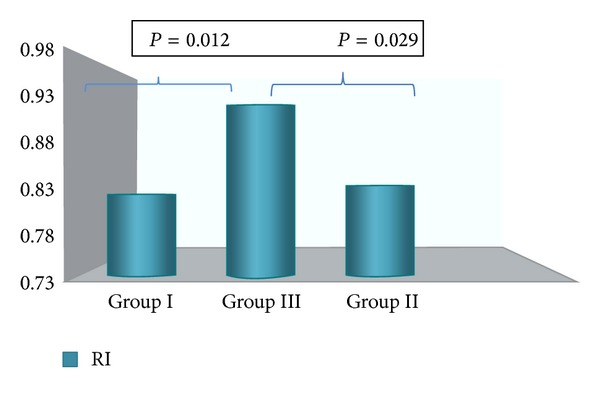
Mean RI values at six months of follow-up (intergroup analysis). A significant difference was observed between Group I (normal cycling women), Group II (amenorrheic), and Group III (prolonged bleedings).

The mean PI and mean RI in the spiral arteries were significantly lower in Group I and Group III compared to Group II (Figures 3 and 4). A significant difference was observed also when comparing the mean PI and mean RI values before and after insertion in Group III.

## 4. Discussion

The present study was designed to test the hypothesis that different bleeding patterns observed after placement of a LNG-IUS may be caused by a different local effect of the system.

Back in 1998, Järvelä et al. [24] conducted a study to measure PI in uterine arteries of women bearing a Cu-IUD and found no significant changes in blood flow after the insertion of the device either during menstruation or in the midluteal phase. At the same time, in women with device-induced dysmenorrhea, a decrease in PI was observed, leading to the conclusion that—overall—the Cu-IUD does not induce any major changes in the resistance of the uterine artery blood flow. In 2006, Jiménez et al. [25] repeated the study and, again, found no significant changes in subendometrial PI and RI, with an endometrial thickness that is lower before than after Cu-IUD insertion.

With regard to the effect of the LNG-IUS on uterine arteries Doppler pulsatility patterns results have been contradictory. In 1995, Pakarinen et al. [26] found no changes in PI before and after a 3-month use of the LNG-IUS, in 10 women utilizing the system for contraceptive purposes, while Järvelä et al. [27] concluded that the system increases uterine arteries impedance to blood flow during the midluteal phase, a phenomenon that correlates with LNG serum concentrations and a concomitant decrease in serum progesterone levels. The same authors [28] observed in postmenopausal women medicated with transdermal estradiol followed, after one month, by placement of an LGN-IUS a decrease in mean uterine artery PI 1 month after transdermal estradiol treatment; however, the LNG-IUS induced an increase in PI, resulting, at the end of 6 months, in a PI that did not differ significantly from the pretreatment level.

It has been postulated that the progestational effect of the system could induce a change in the subendometrial vascularization. Comparing subendometrial flow in women using the Cu-IUD with those bearing the LNG-IUS, Zalel et al. [29] reported a significant reduction in subendometrial flow in spiral arteries in 75% of the LNG system users and none in the Cu-IUD-bearing subjects. At the same time, Doppler flow in the cervical branch of the uterine artery did not reveal any changes between the groups. They interpreted these observations as proof of a local progestational effect on the endometrium with no change in the blood flow in the uterine artery. In 2008, Jiménez et al. [30] carried out a similar comparison and found no significant differences in subendometrial vascularization between the groups, with an increase in PI and RI variability (before and after) and a reduction in endometrial thickness in LNG-IUS users. Finally, Haliloglu et al. [31] compared two groups of 60 women, each bearing a Cu- or a LNG-releasing system, before insertion and 1 year after insertion. They observed no change in Cu-IUD users, whereas at one year RI was significantly higher in LNG-IUS users. The PI was also increased at 1 year at a nonsignificant level. Endometrial thickness was also significantly decreased in the postinsertion period in women with LNG-IUS.

In the present study, the LNG-IUS not only altered endometrial thickness, but—in women with prolonged bleedings—also significantly changed uterine artery blood flow. Indeed, mean PI and mean RI of the spiral arteries were significantly lower in normal cycling and in amenorrheic women when compared to women with prolonged bleedings. This difference persisted also when comparing mean PI and mean RI values before and after insertion with the heavy bleeding group.

With regard to uterine arteries' resistance index in women using the LNG-IUS, an older study [29] reported no changes, whereas a more recent one showed an increase [32]. An explanation of these conflicting results may be provided by the present study where no increased RI value was found in amenorrheic women, whereas a significant increase was observed in women with prolonged bleedings.

Mention should be made of the fact that our study design inevitably entailed a selection bias in women with heavy menstrual bleedings. For this reason, we are currently conducting a new trial including also women without HMB.

In conclusion, while we confirmed what was already reported on the effect of the LNG-IUS on endometrial thickness, we also found a significant alteration in uterine artery blood flow, as evidenced by the RI and PI variability, but only in women with prolonged bleedings. This can be a function of a varying local effect of the system.

Further studies are needed to confirm these results in order to better counsel women and reduce discontinuation rates.

## Figures and Tables

**Table 1:** Women clinical and gynecological characteristics.

	Number	%
Age		
<30	4	12.5
30–40	16	50.0
>40	12	37.5
Parity		
Nulliparous	4	12.5
Parous	28	87.5
Patterns of menstrual bleeding		
PBAC SCORE*	338 (±182 SD**)	NA
Previous contraceptive method used		
Oral hormonal	12	37.5
Intrauterine (IUD***)	5	15.6
Barrier (condom)	15	46.8

*PBAC: Pictorial Blood Assessment Chart Score

**SD: Standard deviation

***IUD: intrauterine device

**Table 2:** Power Doppler analysis, endometrial thickness and uterine volume evaluation at baseline and after 6 months follow up. *P* refers to intragroup analysis.

	Group I (*N* = 8)			Group II (*N* = 16)			Group III (*N* = 6)		
	Baseline	6 months	*P*	Baseline	6 months	*P*	Baseline	6 months	*P*
Uterine arteries Mean PI	1.74 ± 0.49	2.02 ± 0.47	0.15	1.82 ± 0.62	1.73 ± 0.27	0.89	1.76 ± 0.52	2.53 ± 0.33	**0.0001**
Uterine arteries Mean RI	0.79 ± 0.06	0.82 ± 0.08	0.27	0.78 ± 0.03	0.83 ± 0.09	0.17	0.77 ± 0.06	0.92 ± 0.09	**0.0001**
Endom.thickness	5.61 ± 2.89	5.82 ± 1.77	0.81	6.1 ± 3.21	4.2 ± 1.48	0.11	5.72 ± 1.78	7.76 ± 1.17	**0.01**
Uterine Volume	92.96 ± 30.5	92.56 ± 31.0	0.97	95.2 ± 42.5	94.24 ± 40.6	0.94	98.85 ± 49.1	97.45 ± 38.1	0.96
